# Walking Speed Can Be Modulated on an Adaptive Split-Belt Treadmill

**DOI:** 10.3390/app16189175

**Published:** 2026-09-16

**Authors:** Rucha Kulkarni, Barry Bodt, Jill S. Higginson

**Affiliations:** 1 Department of Biomedical Engineering, University of Delaware, Newark, DE 19716, USA; 2 Biostatistics Core, College of Health Sciences, University of Delaware, Newark, DE 19716, USA; 3 Department of Mechanical Engineering, University of Delaware, Newark, DE 19716, USA

**Keywords:** propulsion, gait, adaptive treadmill, validation

## Abstract

Adaptive treadmills (ATMs) that change speed based on the users' gait mechanics can allow for healthy stride-to-stride variability observed during overground walking while maintaining the benefits of traditional treadmill training for gait rehabilitation. To enable unilateral modifications, the purpose of this study was to develop and validate an adaptive split-belt treadmill (sATM) that updates the speed of each belt of the treadmill based on the user's propulsion and position to enable preferential targeting of unilateral gait mechanics. Fourteen young, healthy participants completed five trials at their comfortable walking speed on an existing tied-belt adaptive treadmill (ATM) and sATM with unilateral and bilateral modifications. The participants maintained similar propulsion and belt speeds between treadmills and belts for bilateral modifications. As a group, participants showed no change in propulsion and differences in walking speed for the unilateral modification, demonstrating successful enabling of unilateral modifications. Our results suggest that the sATM may require further tuning to encourage unilateral control strategies. The present study provided a proof of concept of a novel treadmill controller that can enable unilateral modifications, performed technical validation by demonstrating equivalence to an existing tied-belt ATM, and explored unilateral propulsion targeting to demonstrate unilateral modifications.

## 1. Introduction

Walking speed is often used as a marker of healthy gait [1] and community ambulation in older adults with neurological conditions like stroke [1,2]. It is heavily influenced by propulsion (push-off), which can be defined as the positive work performed by the limb to accelerate the body forward [3,4]. For people with post-stroke hemiparesis, the weakened muscles on one side of the body reduce the propulsion forces generated by the paretic (affected) limb, which contributes to a decreased walking speed and affects community ambulation [5].

Treadmill training is commonly used in gait rehabilitation for stroke survivors as it enables high repetition of movement in a safe and controlled environment [2,6]. However, conventional treadmills operate at fixed speeds, which limits any instantaneous changes in speed and propulsion that we might observe in overground walking. Previous rehabilitation paradigms have been limited to fixed-speed treadmills, which limits stride-to-stride variability that is essential for motor learning [7]. Overall, traditional treadmill training shows mixed efficacy and limited improvement in walking speed and propulsion [8].

Split-belt treadmills have been developed such that the walking surface is composed of two belts with speeds set independently. Using this tool, a series of studies has been conducted with the speed of one limb set to twice the other and have shown to improve spatiotemporal gait characteristics in stroke survivors [9,10]. However, the speed differential is controlled by a clinician and may lead to sub-optimal rehabilitation conditions. Furthermore, split-belt treadmills have also been restricted to fixed speeds, which limit stride-to-stride variability (a characteristic of overground walking) and prevent targeting of specific gait kinetics like propulsion. This may play a role in the retention and limited transfer of adapted gait mechanisms from split-belt treadmills to overground walking, which is the ultimate goal of walking rehabilitation [11].

To allow for stride-to-stride variability while retaining any benefits of treadmill training, adaptive treadmills (ATMs) that update speed based on the user gait mechanics (user-driven) have been developed [12,13]. Early treadmill control schemes involved user position on the treadmill or spatiotemporal gait characteristics, and did not consider the relationship between propulsion and walking speed [13]. Recently developed ATMs update speed based on propulsion, step length, and the user's position on the treadmill relative to the treadmill center [12,14]. Studies have shown that healthy individuals walk on an ATM at a self-selected speed closer to overground walking compared to fixed-speed treadmills, suggesting that ATMs may be more similar to overground walking [12]. However, on average, stroke survivors do not seem to increase their self-selected speed on the ATM compared to fixed-speed treadmills [14]. Previous work has also demonstrated that healthy individuals can increase bilateral propulsion to maintain a consistent walking speed on these ATMs with increased propulsion demand (increasing the control parameter for propulsion) [12]. However, to date, these ATMs restrict both limbs to the same speed and thus do not target unilateral gait mechanics. Therefore, the purpose of this study was to develop and validate an adaptive split-belt treadmill (sATM) to enable preferential targeting of unilateral gait mechanics. Specifically, this study aimed to provide a proof of concept of a novel treadmill controller that can enable unilateral modifications, perform technical validation by demonstrating equivalence to an existing tied-belt treadmill, and explore unilateral propulsion targeting to demonstrate unilateral modifications.

The objective of this study was to determine how healthy young adults modulate speed on the novel adaptive split-belt treadmill. Specifically, we aimed (1) to establish the equivalence of the sATM to a previously developed tied-belt treadmill and determine the effect of (2) increased bilateral and (3) increased unilateral demand on the sATM. We hypothesized that: (1) when the control parameters were equal on both belts on the split-belt treadmill and equal to that of a tied-belt treadmill developed by Pariser et al. (2022) [12], there would be no difference in propulsion and belt speed between treadmills; (2) when the control parameter for propulsion (propulsion demand) was increased equally on both belts, propulsion would increase to maintain belt speed; and (3) when propulsion demand was increased for just one belt on the split-belt treadmill, propulsion on the Targeted belt would increase with no difference in belt speed.

## 2. Methods

In this study, participants performed walking trials on a previously developed adaptive tied-belt treadmill and a novel adaptive split-belt treadmill to establish equivalence between the treadmills and to understand the effects of modifying the treadmill. Here, we first describe the novel controller for the adaptive split-belt treadmill and then describe the experimental design and analyses.

### 2.1. sATM Controller

The previous implementation of the adaptive tied-belt (Tied) treadmill updates the speed of both belts based on the current speed, the intermediate speeds due to the average propulsion impulse (net anterior impulse) and the average step length of both limbs, and the position of the person's center of mass (COM) relative to the treadmill center [12]. Intermediate speeds are calculated during swing phase using data from the immediately previous stance phase, and represent the belt speed if only the respective metric determined the overall speed [12].

For application to the split-belt adaptive treadmill (Split), the updated speed of each belt $\left(v_{i+1}\right)$ is calculated independently at each step based on the current speed $\left(v_{i}\right)$, the intermediate speed due to propulsion (net anterior–posterior impulse of the respective limb) $\left(v_{PI}\right)$, and the position of the person's center of mass relative to the treadmill center $\left(COM_{\text{pos}}\right)$ (Figure 1A):

$$v_{i+1}=v_{i}+\gamma v_{PI}-\frac{\gamma }{25}v_{i}+\beta \times \text{sign}\left({\text{COM}}_{\text{pos}}\right)\times \text{CO}{M_{pos}}^{2}$$

where $v_{PI}=\frac{\text{Net AP GRF Impulse of the respective limb}}{\text{Body weight}}(\text{m}/\text{s})$ and $\frac{\gamma }{25}$ is a tuned value from pilot testing.

Thus, increasing or decreasing propulsion increases or decreases the overall belt speed, respectively. Similarly, moving forward (increasing the center of mass position) relative to the treadmill center increases speed and moving backwards decreases speed.

The peak propulsive force was also calculated in real time as the maximum anterior ground reaction force (AGRF) per stride. Peak AGRF asymmetry was then calculated as the difference of the contralateral and ipsilateral peak AGRF divided by the sum.


$$AGRF_{\text{asymm}}=\frac{\text{Peak}\;AGRF_{\text{contralateral}}-\text{Peak}\;AGRF_{\text{ispilateral}}}{\text{Peak}\;AGRF_{\text{contralateral}}+\text{Peak}\;AGRF_{\text{ispilateral}}}$$

To promote healthy gait symmetry, when peak AGRF asymmetry $\left(AGRF_{\text{asymm}}\right)$ was less than 5% [15,16], the updated belt speed was calculated using the intermediate speed due to propulsion (net anterior–posterior impulse) averaged over both limbs:

$$v_{i+1}=v_{i}+\gamma v_{PI,avg}-\frac{\gamma }{25}v_{i}+\beta \times \text{sign}\left(COM_{pos}\right)\times {COM_{pos}}^{2}$$

where $v_{PI,\text{avg}}=\frac{\text{Average Net AP GRF Impulse of both limbs}}{\text{Body weight}}(\text{m}/\text{s})$.

For peak AGRF asymmetry $\left(AGRF_{\text{asymm}}\right)$ greater than 5%, a belt speed penalty is imposed based on the level of asymmetry. Essentially, the updated belt speed is decreased by the intermediate speed due to propulsion (net anterior–posterior impulse) averaged over both limbs around this threshold to remove this discontinuity:

$$v_{i+1}=v_{i}+\gamma v_{PI}-\frac{\gamma }{25}v_{i}+\beta \times \text{sign}\left(COM_{\text{pos}}\right)\times {COM_{pos}}^{2}-\frac{\gamma }{25}\times \text{sign}\left(AGRF_{\text{asymm}}\right)\times AGRF_{\text{asymm}}\times v_{\text{PI, avg}}.$$

The updated speed was calculated between heel-strike and toe-off and updated at toe-off of each stride. To ensure smooth belt accelerations, the propulsion gain (γ) was calculated using an exponential function where dt is the time between belt speed updates (i.e., the time from heel-strike to toe-off for each stride) and the position gain (β) is a tuned value from prior work [12]:

$$\gamma =1-e^{-\frac{dt}{\tau }}$$

β=0.5

The value of tau (τ) was set to either 1.5 or 0.7639 to vary gamma, which is described more in Section 2.2. Decreasing tau (τ) (increasing gamma (γ)) requires higher propulsion at each step to maintain the user's self-selected walking speed.

### 2.2. Data Collection

Fourteen adults (24.79 ± 1.82 years, 1.70 ± 0.08 m, 75.86 ± 14.91 kg, and 7 females) participated and self-reported no known neuromuscular injuries or disorders. The University of Delaware Institutional Review Board approved the protocol (IRB: #2162172-1), and participants gave written informed consent and completed a modified physical activity readiness questionnaire. The participants first performed the 10 m overground walking test to determine their preferred overground walking speed. Then, they performed a series of walking trials on an instrumented split-belt treadmill (Bertec Corp., Columbus, OH, USA; 2000 Hz) and real-time data were collected using a motion capture data collection system (Qualisys, Stockholm, Sweden; 100 Hz).

To demonstrate equivalence, we asked participants to walk on the novel adaptive split-belt treadmill (sATM, Split treadmill) and on a tied-belt adaptive treadmill (Tied treadmill) previously developed by Pariser et al. (2022) [12]. The propulsion demand (gain) was varied bilaterally (Normal and Hard conditions) to establish equivalence for normal and increased bilateral propulsion demand and unilaterally (Uneven) to understand the effect of increased unilateral propulsion demand. The participants performed 5 walking trials for 2 min each—Split Normal, Tied Normal, Tied Hard, Split Hard, and Split Uneven—in a pseudo-randomized order (Figure 1B). For the Normal and Hard conditions on the Split treadmill, the propulsive gains of both belts were identical to the Tied Normal and Hard conditions. The Hard conditions $\left(\tau_{\gamma }=0.7639,\gamma_{\text{Hard}}\right)$ had a higher propulsive gain $\left(\gamma_{\text{Hard}}=1.5\times \gamma_{\text{Normal}}\right)$ than the Normal condition $\left(\tau_{\gamma }=1.5,\gamma_{\text{Normal}}\right)$ and required higher propulsion to maintain a comfortable speed, as observed by Pariser et al. For the Uneven condition, one belt was defined as the Targeted side and had a higher propulsive gain $\left(\gamma_{\text{Hard}}\right)$ than the other side $\left(\gamma_{\text{Normal}}\right)$, such that $\gamma_{\text{Targeted}}=1.5\times \gamma_{\text{Non}–\text{targeted}}$. The Targeted side (right or left belt) was randomized for all participants (n = 7 right).

Participants were given up to 10 min of practice time for each of the Normal conditions before performing the respective Normal trial. No practice trials were given for the Hard or Uneven trials. Both Normal trials (Split and Tied) were performed first in random order, followed by the Hard and Uneven trials in random order. The participants were given up to 3 min to achieve a comfortable, steady-state speed and the trial began when they gave verbal indication that they had achieved a comfortable speed. The maximum speed of the treadmill was set to 2 m/s. The participants were allowed to lightly touch the handrails for stabilization and were visually monitored. After each trial, the participants were asked to respond to a survey to assess their perceived: (1) stability, (2) overall comfort, (3) ease of achieving their preferred walking speed, and (4) ease of maintaining their preferred speed. Each metric was measured on a scale of 1–7 where 1 corresponded to very unstable, uncomfortable, or difficult and 7 represented very stable, comfortable, or easy. The participants also performed a washout trial (1 min) of walking on a fixed speed treadmill at 0.1 m/s less than their preferred overground walking speed after each trial.

### 2.3. Data Processing and Analysis

Kinetic and spatiotemporal data were processed using a custom-written MATLAB 2024a (Natick, MA, USA) program. Force data were filtered using a low-pass fourth-order Butterworth filter with a cutoff frequency of 30 Hz. Heel-strike and toe-off events were identified using a 20 N threshold on the vertical ground reaction forces and these gait events determined each gait cycle. We visually inspected gait events to verify event detection and corrected if necessary.

Our primary outcome measures were belt speed and propulsion metrics. The speed of each belt was recorded for each trial and averaged over the duration of each trial. To evaluate propulsion, we calculated peak anterior (AGRF) ground reaction force and impulse. As secondary measures, we calculated the position of participants' COM to determine their position on the treadmill and propulsion symmetry. To evaluate symmetry between limbs, asymmetry for any metric X was calculated as [15]:

$$\frac{X_{R}-X_{L}}{\frac{1}{2}\left(X_{R}+X_{L}\right)}\times 100\%$$

To further understand how modifying the treadmill influenced kinetic and spatiotemporal components of walking, we calculated peak posterior (PGRF) ground reaction force and impulse, net anterior–posterior ground reaction force impulse, stride length, stride time, step length, and step time using force data (Supplementary Materials). We also noted survey results to understand the perception of stability, comfort, and ease of use of the treadmills.

Kinetic measures were normalized to body weight and spatial measures were normalized to height. Each metric was averaged per limb for all gait cycles in each trial.

### 2.4. Statistical Analysis

An a priori power analysis in RStudio 2024 (Posit, Boston, MA, USA) for an equivalence test for propulsive impulse (bounds = 0.05, power = 0.8, std = 0.05) indicated 10 participants. Significance was set at 0.05 for all statistical analyses. Multiple comparisons were corrected using Bonferroni corrections.

#### 2.4.1. Treadmill Equivalence Analysis

To establish equivalence, we first looked at agreement between belts on the Split treadmill. We evaluated only the Split treadmill for walking speed, as the belt speeds are always equal on the Tied treadmill. We evaluated the linear fit between the right and left belt. We then modeled the average speed of the Normal and Hard conditions for each individual with factors of belt and participant to calculate an intraclass coefficient (ICC) of absolute agreement. The ICC of absolute agreement provides an assessment of how similar speeds are between belts [17].

To determine equivalence between treadmills, we performed Two One-Sided Test (TOST) analyses for our primary outcomes (propulsion and speed). The TOSTs were performed for the difference scores for speed, peak AGRF, and AGRF impulse for the Normal and Hard conditions. Difference scores were calculated as the difference between the Tied and Split treadmill with values averaged over the right and left limb. Equivalence bounds for speed were calculated as minimum detectable change values (±0.194 m/s) calculated using a standard error of measurement obtained from prior work [18]. Equivalence bounds for peak AGRF (±0.038 BW) and AGRF impulse (±0.0055 BW·s) were set using standard deviations from prior work using the Tied treadmill [12]. These values provided bounds that are technically meaningful based on normative data from the prior literature.

All equivalence analyses were performed in JMP Pro (JMP^®^, Version 18.0.1, SAS Institute Inc., Cary, NC, USA).

#### 2.4.2. Propulsion Demand Analysis

To determine the effect of bilaterally increased propulsion demand, we looked at both Split and Tied treadmills. We performed repeated-measures two-way ANOVAs for each belt with the factors of treadmill and condition and their interaction for all kinetic and spatiotemporal measures as well as survey scores. We also evaluated limb symmetry for all kinetic and spatiotemporal measures with repeated-measures two-way ANOVA with the factors of treadmill and condition and their interaction. Confidence intervals (95%, CIs) and effect sizes (partial eta-squared) were calculated from the ANOVA for primary (speed and propulsion) and secondary (COM and propulsion symmetry) outcome measures. Additionally, CIs for the symmetry scores were calculated for each trial.

To determine the effect of unilaterally increased propulsion demand (differences between the belts for the Split Uneven condition), we tested differences between belts for primary outcome measures. We tested paired differences for normality using the Shapiro–Wilk test with visual confirmation using normal Q-Q plots. If no serious departures from normality were observed, we ran paired t-tests. Differences departing from normality were tested using the Wilcoxon signed rank test. Confidence intervals (95%, CIs) and effect sizes (Cohen's d) were calculated from the paired t-tests for primary (speed and propulsion) outcome measures.

All propulsion demand analyses were performed in Python (v. 3.11) [19].

### 2.5. Mathematical Simulation

To explore the trends of the adaptive split-belt treadmill, we also ran mathematical simulations of speed using experimental data. A mathematical model of the sATM was applied to anterior ground reaction force (AGRF) data collected from the Normal Tied condition for a representative participant. This simulation established a theoretical framework of controller behavior to explore how changes in inputs affected speed. The parameters of the model were equal to the experimental algorithm parameters ($\gamma =1-e^{-\frac{dt}{\tau }}$ where τ=1.5 or 0.7639 for Normal and Hard conditions, β=0.5). The initial conditions also matched the experiment, where the starting speed was always 1 m/s.

We varied COM position relative to the treadmill center (0, 0.1, 0.2 m) with no changes in the AGRF data, as well as AGRF data (net impulse + 0.001, 0.005, 0.01 BW·s) with a constant COM position relative to the treadmill center (at 0 m) to observe trends in the simulated velocity of the treadmill.

## 3. Results

### 3.1. Treadmill Equivalence

The speeds of both belts showed linear agreement on the Split treadmill (adj. R^2^ = 0.409). A moderate intracorrelation coefficient (ICC) was found for absolute agreement between left and right belts (ICC(2, 1) = 0.73, 95% CI [0.34, 0.91]).

The participants' self-selected walking (belt) speed was equivalent between Tied and Split treadmills for the Normal (Bonferroni-corrected p<0.001) and Hard (Bonferroni-corrected p<0.001) conditions (Table 1; Figure 2A1).

Peak AGRF was equivalent between both treadmills (Tied and Split) for the Normal (Bonferroni-corrected p<0.001) and Hard (Bonferroni-corrected p<0.001) conditions (Table 1). AGRF impulse was also equivalent between the treadmills for the Normal (Bonferroni-corrected p<0.001) and Hard (Bonferroni-corrected p=0.0086) conditions (Table 1; Figure 2B1).

### 3.2. Effect of Increased Propulsion Demand

#### 3.2.1. Bilateral Increased Propulsion Demand

Both belt speeds were significantly higher in the Hard (higher gamma) condition (Figure 2A1, Table 2). Average AGRF impulse on the right belt was significantly higher on the Tied treadmill (Figure 2B1; Table 2) but showed no significant effect of condition. For the left belt, AGRF impulse was higher in the Hard condition (higher gamma) but had no significant effect of treadmill (Table 2). Peak AGRF was significantly higher in the Hard condition for both belts and significantly higher in the Tied treadmill for the right belt but not the left belt (Table 2).

AGRF impulse symmetry showed a statistically significant effect of treadmill but not condition (Table 2). Peak AGRF symmetry showed a significant effect of treadmill but not condition (Table 2).

The COM position was significantly correlated between right and left strides (adj. R^2^ = 0.99). There was a significant interaction between treadmill and condition on COM position (F(1,13) = 250.184, p<0.001). The participants' COM was significantly more forward in the Hard condition for both Tied (Bonferroni-corrected p=0.0027) and Split (Bonferroni-corrected p=0.027).

In the few cases where the residual normality assumption for the ANOVAs was not met, the recovery action of outlier removal did not alter which effects were significant.

#### 3.2.2. Unilateral Increased Propulsion Demand

For the Split Uneven condition, paired differences for belt speed, AGRF impulse, and peak AGRF were normal (Shapiro–Wilk test p>0.05). Belt speed was significantly different between belts (p=0.0417, 95% CI = [−0.26, −0.01], Cohen’s d = 0.57), where the Targeted belt speed was slower than the Non-Targeted speed (Figure 2A2, Table 1). AGRF impulse (p=0.583, 95% CI = [−0, 0], Cohen’s d = 0.077, Figure 2B2) and peak AGRF (p=0.855, 95% CI = [−0.02, 0.02], Cohen’s d = 0.072) did not show a significant difference between belts (Table 1).

### 3.3. Survey Results

Survey data were compared between all five conditions (Table 3). The participants reported that it was slightly easier to achieve and maintain speed and they felt more comfortable and stable on the Tied ATM. The participants also reported that it was easier to achieve and maintain speed and felt more comfortable and stable in the Normal condition than the Hard condition for both treadmills as well as the Split Uneven condition.

### 3.4. Mathematical Simulation

The simulated belt speed responded to variations in COM position as well as net anterior–posterior impulse (Figure 3). At the center of the treadmill $\left(COM_{\text{pos}}=0\right)$ with the original impulse data from a representative participant (experimental $v_{PI}$), the simulated speed declined with the number of strides (Figure 3A). This indicates that the impulse was lower than required to maintain a speed of 1 m/s at that position, and the participant was likely slightly closer to the front during the experiment, as moving forward on the treadmill would increase speed. Increasing the COM position relative to the treadmill center (increasing $COM_{\text{pos}}$) corresponded to an increased simulated belt speed. Similarly, an increase in net impulse (increase in $v_{PI}$) showed an increase in belt speed when the COM position was held constant at the treadmill center $\left(COM_{\text{pos}}=0\right)$.

## 4. Discussion

We developed and validated a novel, adaptive split-belt treadmill. We hypothesized that propulsion and walking speed on the novel sATM would be equivalent to a previously developed Tied adaptive treadmill, and further that the sATM could be bilaterally and preferentially unilaterally weighted to encourage higher propulsion while maintaining walking speed. Our findings partially support our hypothesis: participants maintained similar propulsion and walking speed between treadmills and belts. The participants also increased propulsion and walking speeds with increased bilateral propulsion demand. Interestingly, while participants maintained significantly different walking speeds between belts when the sATM was preferentially unilaterally weighted, there was no significant difference between belts for propulsion on average. However, a few participants increased unilateral propulsion to maintain similar walking speeds, while others showed no change in propulsion with slight differences in walking speed. These results suggest that the response to unilateral weighting was user-specific, and the sATM could be further tuned to individual responses to encourage unilateral changes.

The present study provided a proof of concept of a novel treadmill controller that can enable unilateral modifications with a preliminary validation in healthy, young adults before application in clinical populations.

### 4.1. Treadmill Equivalence

Our study demonstrated equivalence for speed and propulsion between the novel adaptive split-belt treadmill and a previously developed tied-belt treadmill [12].

The participants maintained similar speeds between belts on the Split treadmill, shown by a moderate agreement between belt speeds. It is possible that participants did not perceive any differences between belts during the trials. A total of 61% (17/28) of Split trials were in the healthy gait asymmetry range $\left(AGRF_{\text{asymm}}\leq 5\%\right)$, suggesting that the sATM controller on average used a $v_{PI}$ averaged over both limbs. Furthermore, previous work shows that healthy participants perceive belt speed differences during split-belt treadmill walking after a certain threshold of difference (95% CI: [0.025, 0.105]) is achieved [20]. Post hoc analysis of the perception threshold (absolute belt speed difference/sum of belt speeds) showed that the confidence interval of the perception threshold for all participants in both Split Normal ([0.033, 0.093]) and Split Hard (higher gamma, [0.035, 0.078]) conditions were within the aforementioned confidence intervals.

We found that participants maintained their walking speed between Tied and Split treadmills. Propulsion (peak AGRF and AGRF impulse) was also consistent between treadmills and belts in the Normal and Hard conditions. While peak AGRF symmetry and AGRF impulse symmetry showed a significant effect of treadmill, symmetries for each condition were within healthy gait symmetry levels (±15.6, ±18.4, respectively) [15]. Therefore, we believe these bilateral differences are not meaningful. Overall, this equivalence analysis was meaningful for “typical” movement, as our equivalence bounds were derived from normative values.

### 4.2. Increased Propulsion Demand

The participants had a higher self-selected speed in the Hard conditions (higher gamma) compared to the Normal conditions. While participants did not increase their AGRF impulse consistently between conditions, they were significantly closer to the front on the treadmill in the Hard condition for both treadmills. This suggests that participants modulated their position on the treadmill instead of AGRF impulse. Additionally, the participants were instructed to walk at a comfortable speed, not their overground speed, and may not have sensed an “uncomfortable” change in walking speed between the Hard and Normal conditions.

The participants increased their peak AGRF for the Hard conditions on both treadmills, suggesting that they were pushing off harder. These results indicate that by increasing the propulsion gain, we can encourage volitional bilateral changes in propulsion on the sATM. Peak AGRF is a good indicator of propulsion during walking in individuals post-stroke [21]; therefore, our results suggest that this could potentially be a useful tool to investigate peak AGRF modulation in stroke survivors.

Mathematically, we can demonstrate that increased propulsion is required for increased gamma (increased propulsion demand), which is similar to previous Tied ATM work [12]. If we assume that users maintain similar positions on the treadmill at each stride, the change in speed between strides $\left(v_{i+1}-v_{i}\right)$ is driven primarily by the difference between the intermediate speed due to propulsion and the penalty factor based on the current belt speed $\left(\gamma v_{PI}-\frac{\gamma }{25}v_{i}\right)$. Therefore, under such conditions, to maintain the exact same speed on the treadmill regardless of gamma, the participant would need to maintain $v_{PI}=\frac{1}{25}v_{i}$. For human walking, the operational range of the treadmill lies within typical values of $v_{PI}$ (net impulse) and belt speed from the participants, which lead to $v_{PI}<\frac{1}{25}v_{i}$, thus driving a slow decay of the belt speed, which is then regulated by the propulsion demand term (gamma). Therefore, for a higher gamma, the speed decay would be faster, which requires higher intermediate speed due to propulsion $\left(v_{PI}\right)$ to maintain speed. While the speed would increase faster if $v_{PI}>\frac{1}{25}v_{i}$ for a higher gamma, this condition lies outside the typical operational range, and participants do not maintain increased $v_{PI}>\frac{1}{25}v_{i}$ for more than a few gait cycles. Thus, this would only last until equilibrium is achieved, beyond which if the speed needs to be altered, the controller would once again fall under the previous conditions $\left(v_{PI}<\frac{1}{25}v_{i}\right)$ and require increased propulsion. Therefore, although increased propulsion might not be constantly applied throughout all strides in the Hard condition, participants would require consistently higher propulsion on average throughout the trial.

Despite different propulsion weights, the Split Uneven condition did not show significant differences in propulsion (peak AGRF and AGRF impulse) on the Targeted limb on average. It is possible that this led to the speed difference between the belts, where the Targeted belt speed was lower than the Non-Targeted side. Since the propulsion demand was greater on the Targeted side, without an increase in their propulsion, the belt speed is slower. However, on an individual level, a few participants increased their propulsion on the Targeted limb and achieved a smaller difference between belt speeds. Maintaining speed between belts would require a sufficient increase in unilateral propulsion. If this is not achieved, then speeds will differ. Our results demonstrate that the controller performs as expected theoretically with unilateral modifications. However, our hypothesis that participants would increase propulsion to maintain speeds between belts was not supported.

While a peak AGRF asymmetry of less than or equal to 5% led to the use of an average bilateral net anterior–posterior impulse in the mathematical equation of the controller, the propulsion demand (gain) was different for both belts. The bilateral average propulsion was only applied to support healthy gait symmetry but did not fundamentally change the controller equation. Therefore, we do not believe it weakened the effect of the unilateral intervention.

Furthermore, net anterior–posterior impulse was higher on the Targeted limb and the PGRF impulse was higher on the Non-Targeted limb (higher braking) (Supplementary Materials). As net impulse is a combination of propulsive and braking impulse, our results of increased braking impulse on the Non-Targeted side and no significant differences in propulsive impulse may have contributed to a higher net impulse on the Targeted side. This may suggest that participants were modulating braking to decrease speed on the Non-Targeted side as belt speed is modulated by net impulse. However, the significant difference in belt speeds suggests that this increased net impulse by modulating the braking impulse was not enough. Additionally, absolute values of both braking and propulsive impulse increase with an increase in speed [22]. It is therefore possible that participants modulated propulsive and braking impulses differently on both sides of the treadmill—due to either speed (Non-Targeted side) or propulsion demand (Targeted side)—but we cannot conclude that increased braking was an essential part of their control strategy. Additionally, participants did not practice the Split Uneven condition and may have struggled to accommodate to the difference between belts. These results suggest that the response to preferential weighting of unilateral propulsion is user-specific and may need to be tuned to individual responses to encourage a significant increase.

The simulation results demonstrate how the speed of each belt can be changed by modulating the COM position or net anterior impulse. We verified that the sATM controller algorithm behaves as anticipated by performing speed simulations using data collected from a participant. These results show how participants may have implemented different strategies to maintain comfortable walking speeds on the treadmill.

The survey results indicate that while participants perceived that achieving and maintaining their preferred speed was easier on the Tied ATM compared to the sATM for the Normal condition, there was no significant difference between treadmills for the Hard conditions. Additionally, the Split Normal and Uneven conditions were similar to the Hard conditions in all survey responses. This suggests that while participants may have found it easier to use the Tied ATM, they were able to control their walking speed on all sATM conditions with relative ease.

In the current work, users tended to modulate their position on the treadmill instead of propulsion to control the speed of the treadmill. Previous work has suggested that modifying the importance of position on the treadmill may result in changes in net impulse on the Tied ATM [23]. While drastically reducing the importance of position increased difficulty in achieving and maintaining walking speeds, it did not affect the comfort and stability of use of the Tied ATM. Therefore, future work should explore modifying the COM term in the sATM controller to reduce reliance on the modulation of position on the treadmill while maintaining ease of use. It is also possible that the light use of the handrails could be a potential confounding factor for increased propulsion as, biomechanically, the use of handrails can provide assistance in increasing propulsive impulse. However, we believe that our instructions of “light touch of the handrails” and visual inspection minimized any effects. Additionally, although our a priori power analysis was based on achieving 80% power, this study had a limited sample size of 14 able-bodied, young participants and future work should look at a larger sample as well as clinical populations to establish the generalizability of these results.

## 5. Conclusions

The present study demonstrates that walking speed can be modulated on an adaptive split-belt treadmill. In this work, we developed and validated an sATM that can be modified unilaterally. Future work will explore different combinations of the propulsion gains on the sATM in the Split Uneven condition to determine gains that promote increased unilateral propulsion in healthy and post-stroke individuals.

## Supplementary Material

Supplementary Materials

**Supplementary Materials:** The following supporting information can be downloaded at: https://www.mdpi.com/article/10.3390/app16189175/s1, File Supplemental Materials_SATM (Text): analysis of supplemental kinetic and spatiotemporal variables (Table S1: Secondary kinetic and spatiotemporal metrics; Table S2: Statistical analysis results for Normal and Hard trials); File Data_WalkingSpeedModulatedOnSATM (Processed Data): anthropometric and processed data from this study; File SupplementalData_WalkingSpeedModulatedOnSATM (Processed Data): processed data described in the supplemental text file for this study.

## Figures and Tables

**Figure 1.**
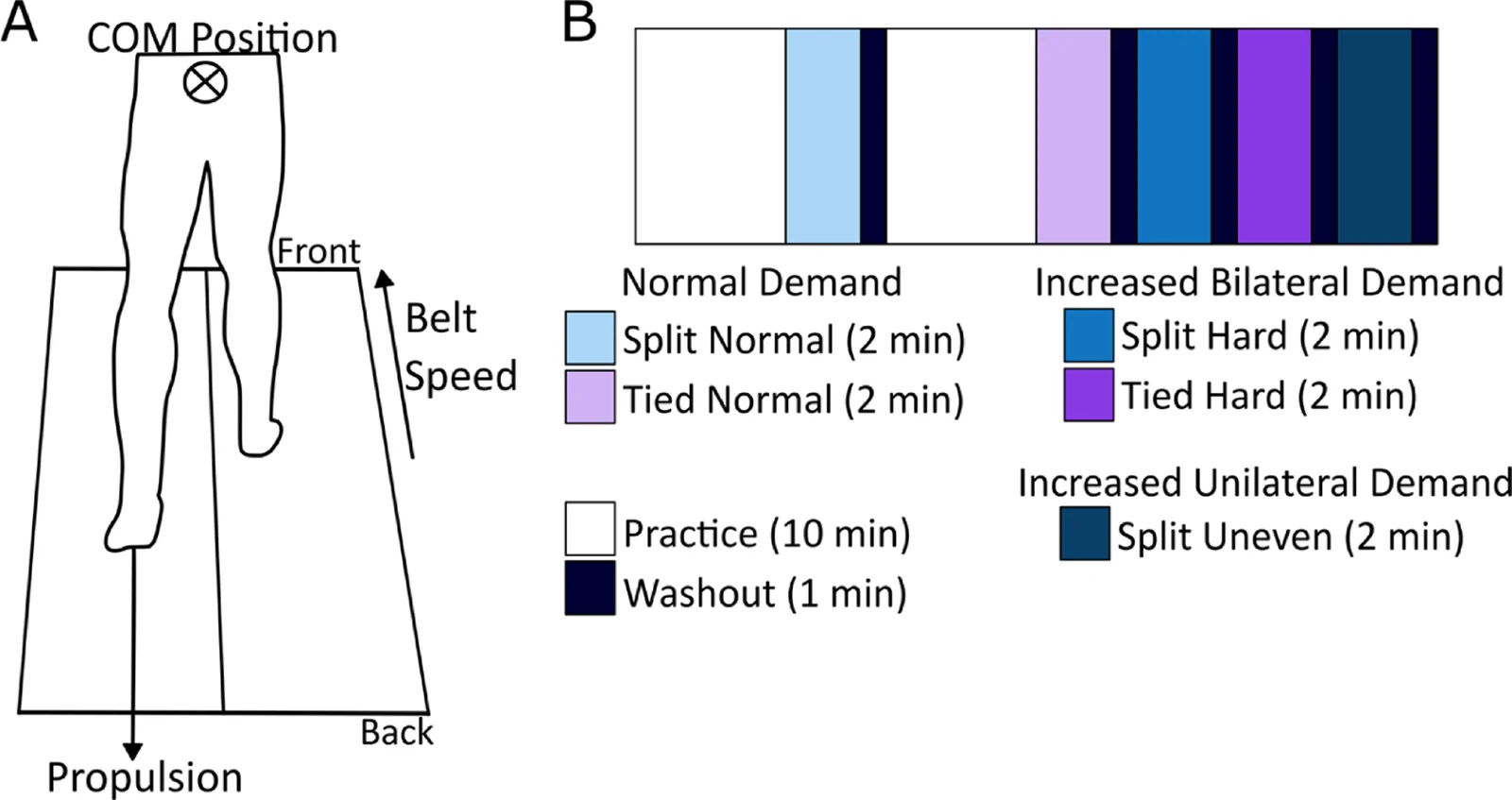
(**A**) Experimental set-up. An illustrative sketch of a participant on the sATM. (**B**) Experimental protocol. The participants performed 5 trials on the Tied ATM and Split ATM.

**Figure 2.**
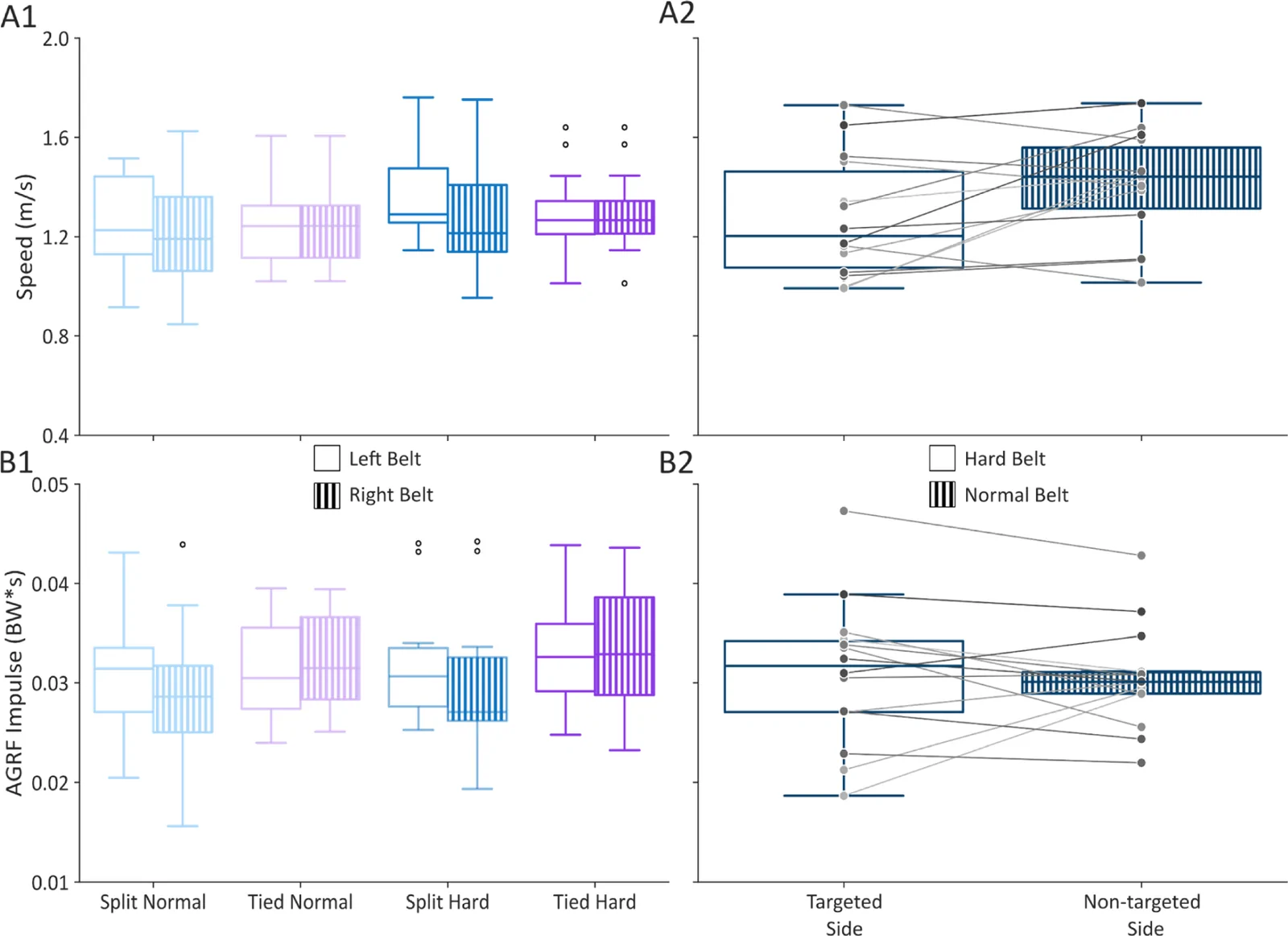
(**A1**) Walking speed; (**B1**) AGRF impulse. Box plots represent data from all participants for the right and left belt for Normal and Hard conditions. (**A2**) Walking speed; (**B2**) AGRF impulse. Box plots represent data from all participants for the belt with normal propulsion demand (Non-Targeted) and increased propulsion demand (Targeted) for the Split Uneven condition. Line plots represent data from individual participants for the Targeted and Non-Targeted belts in the Split Uneven condition.

**Figure 3.**
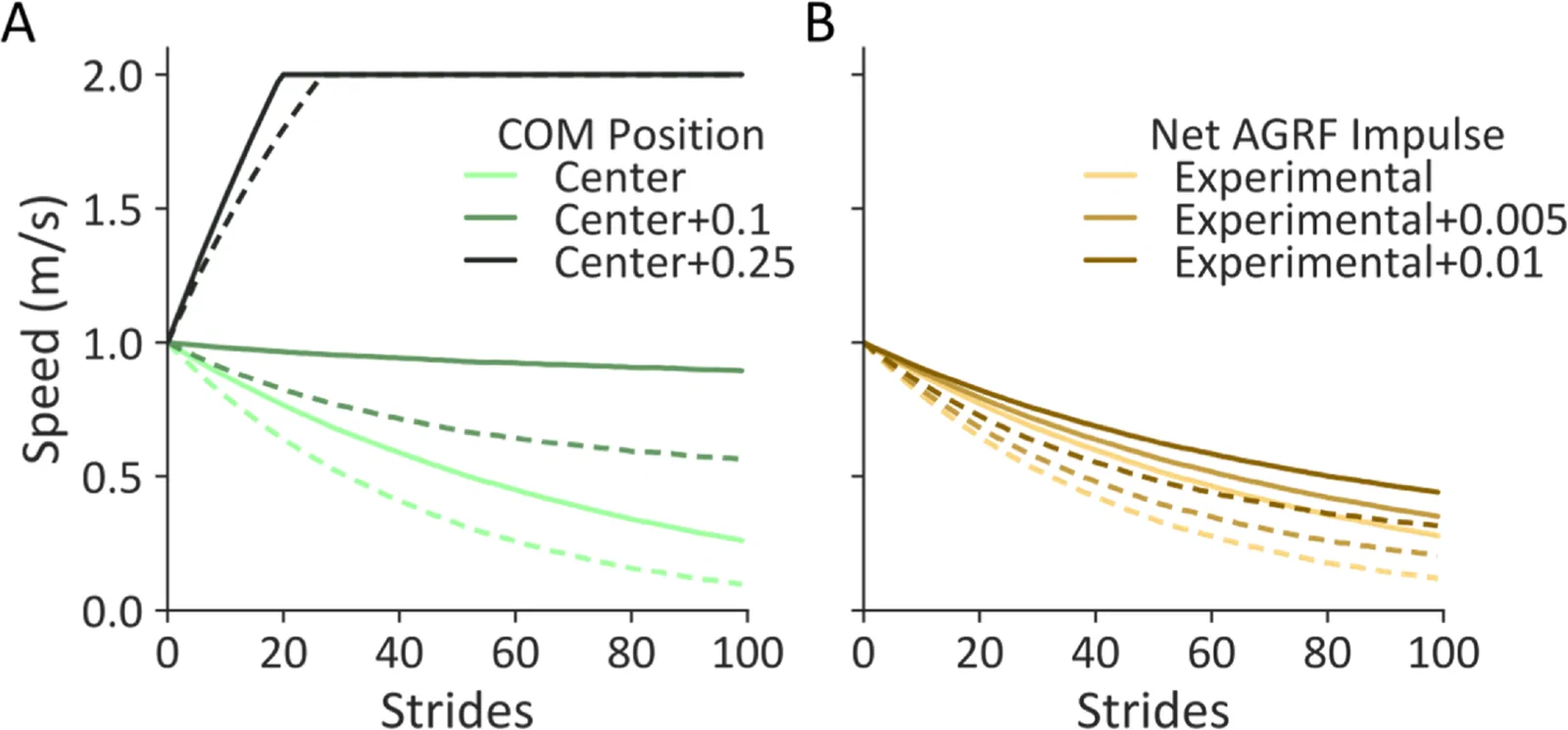
Simulation Results: (**A**) Varying Position: belt speed was simulated using the control equation for different center of mass (COM) positions relative to the center of the treadmill with the net AGRF impulse of a representative participant. (**B**) Varying Net Impulse: belt speed was simulated using the control equation by varying the net AGRF impulse (by incrementing experimental data) of a representative participant. Solid lines depict the Split Normal condition, and dashed lines depict the Split Hard condition.

**Table 1.** Kinetic metrics and COM position (mean ± SD) for all walking trials.

Treadmill	Tied		Split		Split Uneven	
	Normal	Hard	Normal	Hard	Targeted Side	Non-Targeted Side
Belt Speed (m/s)	R: 1.24 ± 0.16	R: 1.3 ± 0.16	R: 1.22 ± 0.23	R: 1.28 ± 0.23	1.28 ± 0.23	1.41 ± 0.21
	L: 1.24 ± 0.16	L: 1.3 ± 0.16	L: 1.26 ± 0.18	L: 1.38 ± 0.19		
Peak AGRF (%BW)	R: 20.91 ± 3.81	R: 22.62 ± 4.81	R: 19.61 ± 5.33	R: 20.87 ± 5.77	21.65 ± 5.77	22.05 ± 5.17
	L: 20.97 ± 4.64	L: 22.67 ± 5.45	L: 20.84 ± 4.88	L: 22.67 ± 5.38		
AGRF Impulse (%BW·s)	R: 3.20 ± 0.49	R: 3.33 ± 0.62	R: 2.91 ±0 .69	R: 2.95 ± 0.69	3.09 ± 0.71	3.05 ± 0.50
	L: 3.13 ± 0.51	L: 3.29 ± 0.55	L: 3.07 ± 0.58	L: 3.16 ± 0.56		
Center of Mass (m)	R: 0.57 ± 0.019	R: 0.79 ± 0.009	R: 0.62 ± 0.013	R: 0.65 ± 0.046	0.64 ± 0.014	0.64 ± 0.014
	L: 0.57 ± 0.019	L: 0.79 ± 0.009	L: 0.62 ± 0.013	L: 0.65 ± 0.045		

**Table 2.** Statistical analysis results (F-statistic, p-values, confidence intervals, and partial eta-squared effect size) for Normal and Hard trials.

		Treadmill (Tied vs. Split)		Condition (Normal vs. Hard)		Interaction (Treadmill × Condition)	
		Right Belt	Left Belt	Right Belt	Left Belt	Right Belt	Left Belt
Belt Speed (m/s)	F(1,13)	0.347	3.699	5.569	35.299	0.0640	1.382
	p	0.57	0.076	0.0346	<0.001	0.804	0.261
	CIs	Tied: 1.16–1.38 Split: 1.14–1.36	1.17–1.36 1.22–1.42	Normal: 1.13–1.33 Hard: 1.18–1.39	1.16–1.34 1.25–1.43	-	-
	Effect size	0.026	0.22	0.29	0.73	0.0049	0.096
Peak AGRF (%BW)	F(1,13)	6.557	0.0239	6.839	25.247	0.275	0.0180
	p	0.023	0.879	0.021	0.001	0.609	0.895
	CIs	Tied: 0.19–0.25 Split: 0.17–0.23	0.19–0.25 0.19–0.25	Normal: 0.17–0.23 Hard: 0.19–0.25	0.18–0.24 0.20–0.26	-	-
	Effect size	0.34	0.0018	0.34	0.66	0.021	0.0014
Peak AGRF Asymmetry (%)	F(1,13)	15.581		0.134		0.156	
	p	0.0017		0.719		0.699	
	CIs	Tied: (−3.58)–4.31 Split: (−11.95)–(−4.06)		Normal: (−7.73)–1.18 Hard: (−8.82)–0.093		-	
	Effect size	0.55		0.010		0.012	
AGRF Impulse (%BW·s)	F(1,13)	12.88	3.731	0.893	5.008	0.562	0.515
	p	0.0033	0.075	0.362	0.043	0.467	0.485
	CIs	Tied: 0.19–0.25 Split: 0.026–0.035	0.029–0.035 0.028–0.034	Normal: 0.027–0.034 Hard: 0.028–0.035	0.028–0.034 0.029–0.035	-	-
	Effect size	0.50	0.22	0.064	0.28	0.041	0.038
AGRF Impulse Asymmetry (%)	F(1,13)	15.957		0.193		0.0140	
	p	0.0015		0.67		0.908	
	CIs	Tied: (−3.75)–6.89 Split: (−12.45)–(−1.81)		Normal: (−7.94)–3.90 Hard: (−9.46)–2.38		-	
	Effect size	0.55		0.015		0.0011	
COM position	F(1,13)	40.580		511.358		250.184	
	p	<0.001		<0.001		<0.001	
	CIs	Tied: 0.67–0.69 Split: 0.62–0.65		Normal: 0.58–0.60 Hard: 0.71–0.73		-	
	Effect size	0.76		0.98		0.95	

**Table 3.** Survey data (mean ± SD) for all walking trials.

Treadmill	Tied		Split		
Condition	Normal	Hard	Normal	Hard	Uneven
Achieve Speed	6.5 ± 0.65	4.21 ± 1.48	5.21 ± 1.31	4.71 ± 1.49	4.43 ± 1.65
Maintain Speed	6.43 ± 0.51	4.86 ± 1.56	4.71 ± 1.38	4.86 ± 1.17	4.21 ± 1.37
Stability	6.64 ± 0.5	5.36 ± 1.39	5.07 ± 1.49	5.14 ± 1.29	4.57 ± 1.79
Comfort	6.86 ± 0.36	5.43 ± 1.28	4.93 ± 1.44	5.29 ± 1.14	4.36 ± 1.55

## Data Availability

The original contributions presented in this study are included in the Supplementary Material. Further inquiries can be directed to the corresponding author.
