# Supplementary material for: Walking Speed Can Be Modulated on an Adaptive Split-Belt Treadmill
Source: Appl Sci (Basel). Author manuscript; Available in PMC 2026 Nov 4. (PMC13730155; doi:10.3390/app16189175)
Supplement: Supplementary file 1 [file NIHMS2217769-supplement-Supplementary_Materials.zip › SupplementalMaterials_SATM.pdf]

SUPPLEMENTAL MATERIALS

We analyzed secondary kinetic and spatiotemporal measures to provide further insight into participants' walking on both treadmills for all conditions (Table S1).

Effect of Bilateral Propulsion Demand

Statistical analysis values are reported in Table S2.

Average PGRF impulse showed a significant effect of treadmill and condition for both belts. PGRF impulse symmetry showed no significant effect of treadmill or condition. Peak PGRF had a significant effect of condition for both belts and treadmill for the right belt. There was no significant effect of treadmill or condition on peak PGRF symmetry. Net anterior-posterior impulse on the left belt showed a significant effect of treadmill and condition, while the right belt had no significant differences. Net anterior-posterior impulse symmetry showed no significant differences between treadmill or condition.

AGRF time showed a significant effect of treadmill and condition on the left belt. PGRF time showed a significant effect of condition on both belts and treadmill on the left belt but not the right. There was no significant effect of treadmill or condition on AGRF time symmetry and PGRF time symmetry. The peak AGRF time was significantly different between conditions for both belts and between treadmills for the left belt but not the right. There was no significant effect of treadmill or condition on the peak AGRF time symmetry. The peak braking time was significantly different between conditions for both belts and between treadmills for the left belt but not right. There was a significant effect of treadmill but not condition on the symmetry of the time of peak braking.

Stride length showed a significant effect of condition on the left belt. Stride length symmetry showed no significant differences. Step length had a significant effect of condition on the right belt and effect of treadmill on the left belt. There was a significant effect of treadmill on step length symmetry. Stride time showed a significant effect of condition on both belts. Stride time symmetry showed no significant differences. Step time was significantly different between conditions for both belts and between treadmills for the right belt. Step time symmetry showed no significant effect of treadmill or condition.

Effect of Unilateral Propulsion Demand

Normality was checked for paired differences using the Shapiro-Wilk test. All measures were normal except for Stride Time (Shapiro-Wilk $p<0.001$)

For Split Uneven, there was no significant difference between belts for PGRF time, peak PGRF time, stride time, or step length. PGRF impulse ($p = 0.025$), peak PGRF ($p = 0.031$), stride length ($p = 0.044$), and step time ($p = 0.032$) were greater for the Non-targeted limb. Net anterior-posterior impulse ($p = 0.030$), AGRF time ($p = 0.001$), and peak AGRF time (0.027) were greater for the Targeted limb.

Table S1. Secondary kinetic and spatiotemporal metrics (Mean ± SD).

Treadmill	Tied		Split		Split Uneven	
	Normal	Hard	Normal	Hard	Non-Targeted Side	Targeted Side
**Peak PGRF (%BW)**	**R:** 19.15±4.95<br>**L:** 19.62±4.57	**R:** 21.63±5.74<br>**L:** 22.34±5.39	**R:** 18.19±5.33<br>**L:** 18.93±4.74	**R:** 19.86±5.42<br>**L:** 21.44±5.12	22.21±6.21	20.75±5.81
**PGRF Impulse (%BW*s)**	**R:** 3.29±0.53<br>**L:** 3.37±0.50	**R:** 3.57±0.57<br>**L:** 3.66±0.61	**R:** 3.08±0.65<br>**L:** 3.19±0.55	**R:** 3.13±0.54<br>**L:** 3.38±0.47	3.48±0.63	3.22±0.56
**Net Impulse (%BW)**	**R:** -0.09±0.25<br>**L:** -0.24±0.25	**R:** -0.24±0.27<br>**L:** -0.36±0.24	**R:** -0.18±0.21<br>**L:** -0.11±0.24	**R:** -0.18±0.44<br>**L:** -0.22±0.27	-0.43±0.27	-0.12±0.22
**AGRF Time (s)**	**R:** 0.32±0.026<br>**L:** 0.32±0.030	**R:** 0.32±0.030<br>**L:** 0.31±0.027	**R:** 0.31±0.031<br>**L:** 0.31±0.019	**R:** 0.31±0.022<br>**L:** 0.30±0.020	0.29±0.020	0.31±0.026
**PGRF Time (s)**	**R:** 0.39±0.037<br>**L:** 0.39±0.031	**R:** 0.37±0.037<br>**L:** 0.38±0.030	**R:** 0.38±0.041<br>**L:** 0.38±0.032	**R:** 0.37±0.043<br>**L:** 0.36±0.034	0.36±0.038	0.37±0.036
**Peak AGRF Time (s)**	**R:** 0.61±0.05<br>**L:** 0.62±0.05	**R:** 0.59±0.04<br>**L:** 0.60±0.04	**R:** 0.60±0.05<br>**L:** 0.59±0.04	**R:** 0.58±0.05<br>**L:** 0.57±0.05	0.56±0.048	0.59±0.51
**Peak PGRF Time (s)**	**R:** 0.13±0.02<br>**L:** 0.13±0.03	**R:** 0.13±0.02<br>**L:** 0.12±0.02	**R:** 0.13±0.02<br>**L:** 0.12±0.02	**R:** 0.12±0.02<br>**L:** 0.11±0.02	0.11±0.025	0.12±0.024
**Stride Length**	**R:** 0.48±0.061<br>**L:** 0.48±0.061	**R:** 0.49±0.058<br>**L:** 0.49±0.057	**R:** 0.46±0.079<br>**L:** 0.47±0.067	**R:** 0.46±0.068<br>**L:** 0.50±0.069	0.87±0.11	0.79±0.13
**Step Length**	**R:** 0.24±0.031<br>**L:** 0.24±0.030	**R:** 0.24±0.029<br>**L:** 0.25±0.029	**R:** 0.23±0.032<br>**L:** 0.23±0.037	**R:** 0.25±0.033<br>**L:** 0.24±0.032	0.40±0.058	0.42±0.051
**Stride Time (s)**	**R:** 1.12±0.07<br>**L:** 1.12±0.07	**R:** 1.09±0.06<br>**L:** 1.09±0.06	**R:** 1.09±0.07<br>**L:** 1.09±0.07	**R:** 1.06±0.06<br>**L:** 1.06±0.06	1.06±0.063	1.06±0.062
**Step Time (s)**	**R:** 0.56±0.03<br>**L:** 0.56±0.04	**R:** 0.54±0.03<br>**L:** 0.55±0.03	**R:** 0.54±0.03<br>**L:** 0.55±0.04	**R:** 0.52±0.03<br>**L:** 0.54±0.04	0.54±0.036	0.52±0.038

Table S2. Statistical analysis results for Normal and Hard trials.

	Treadmill (Tied vs. Split)		Condition (Normal vs. Hard)		Interaction (Treadmill X Condition)	
	**Right Belt**	**Left Belt**	**Right Belt**	**Left Belt**	**Right Belt**	**Left Belt**
**Peak PGRF (%BW)**	F(1,13) = 5.054 p = 0.043	F(1,13) = 1.653 p = 0.221	F(1,13) = 22.331 p < 0.001	F(1,13) = 57.377 p < 0.001	F(1,13) = 1.024 p = 0.330	F(1,13) = 0.062 p = 0.807
**Peak PGRF Asymmetry (%)**	F(1,13) = 2.252 p = 0.157		F(1,13) = 1.768 p = 0.206		F(1,13) = 0.690 p = 0.421	
**PGRF Impulse (%BW*s)**	F(1,13) = 17.095 p = 0.001	F(1,13) = 9.292 p = 0.009	F(1,13) = 6.999 p = 0.020	F(1,13) = 13.124 p = 0.003	F(1,13) = 4.511 p = 0.053	F(1,13) = 0.714 p = 0.
**PGRF Impulse Asymmetry (%)**	F(1,13) = 2.591 p = 0.132		F(1,13) = 0.973 p = 0.342		F(1,13) = 1.799 p = 0.203	
**Net Impulse (%BW*s)**	F(1,13) = 0.017 p = 0.899	F(1,13) = 6.077 p = 0.028	F(1,13) = 1.559 p = 0.234	F(1,13) = 6.198 p = 0.027	F(1,13) = 2.629 p = 0.129	F(1,13) = 0.022 p = 0.883
**Net Impulse Asymmetry (%)**	F(1,13) = 0.832 p = 0.378		F(1,13) = 1.101 p = 0.313		F(1,13) = 0.885 p = 0.364	
**AGRF Time (s)**	F(1,13) = 1.324 p = 0.271	F(1,13) = 5.675 p = 0.033	F(1,13) = 1.263 p = 0.281	F(1,13) = 5.061 p = 0.042	F(1,13) = 0.311 p = 0.587	F(1,13) = 1.588 p = 0.230
**AGRF Time Asymmetry (%)**	F(1,13) = 0.371 p = 0.553		F(1,13) = 2.94 p = 0.110		F(1,13) = 1.962 p = 0.185	
**PGRF Time (s)**	F(1,13) = 1.196 p = 0.294	F(1,13) = 6.148 p = 0.0276	F(1,13) = 13.062 p = 0.003	F(1,13) = 57.053 p < 0.001	F(1,13) = 2.511 p = 0.137	F(1,13) = 0.312 p = 0.586
**PGRF Time Asymmetry (%)**	F(1,13) = 3.901 p = 0.069		F(1,13) = 0.114 p = 0.741		F(1,13) = 0.522 p = 0.483	
**Peak AGRF Time (s)**	F(1,13) = 1.798 p = 0.203	F(1,13) = 8.340 p = 0.013	F(1,13) = 9.190 p = 0.010	F(1,13) = 18.449 p < 0.001	F(1,13) = 0.220 p = 0.647	F(1,13) = 0.168 p = 0.688
**Peak AGRF Time Asymmetry (%)**	F(1,13) = 1.513 p = 0.240		F(1,13) = 0.992 p = 0.337		F(1,13) = 1.189 p = 0.295	
**Peak PGRF Time (s)**	F(1,13) = 2.555 p = 0.134	F(1,13) = 8.700 p = 0.011	F(1,13) = 5.332 p = 0.038	F(1,13) = 12.104 p = 0.004	F(1,13) = 1.091 p = 0.315	F(1,13) = 0.624 p = 0.444
**Peak PGRF Time Asymmetry (%)**	F(1,13) = 10.515 p = 0.006		F(1,13) = 2.292 p = 0.154		F(1,13) = 0.357 p = 0.561	
**Stride Length**	F(1,13) = 4.268 p = 0.059	F(1,13) = 0.870 p = 0.368	F(1,13) = 1.049 p = 0.324	F(1,13) = 13.568 p = 0.003	F(1,13) = 0.385 p = 0.546	F(1,13) = 1.320 p = 0.271
**Stride Length Asymmetry (%)**	F(1,13) = 4.386 p = 0.0564		F(1,13) = 1.074 p = 0.319		F(1,13) = 1.168 p = 0.299	
**Step Length**	F(1,13) = 0.017 p = 0.898	F(1,13) = 5.021 p = 0.043	F(1,13) = 18.307 p < 0.001	F(1,13) = 2.417 p = 0.144	F(1,13) = 1.393 p = 0.259	F(1,13) = 0.133 p = 0.721
**Step Length Asymmetry (%)**	F(1,13) = 7.073 p = 0.020		F(1,13) = 0.095 p = 0.348		F(1,13) = 1.152 p = 0.303	
**Stride Time (s)**	F(1,13) = 4.157 p = 0.062	F(1,13) = 4.091 p = 0.0642	F(1,13) = 11.076 p = 0.005	F(1,13) = 11.470 p = 0.005	F(1,13) = 0.086 p = 0.773	F(1,13) = 0.078 p = 0.785
**Stride Time Asymmetry (%)**	F(1,13) = 1.162 p = 0.301		F(1,13) = 3.74 p = 0.075		F(1,13) = 0.222 p = 0.646	
**Step Time (s)**	F(1,13) = 8.076 p = 0.014	F(1,13) = 1.058 p = 0.322	F(1,13) = 12.389 p = 0.004	F(1,13) = 6.066 p = 0.029	F(1,13) = 0.527 p = 0.481	F(1,13) = 0.009 p = 0.927
**Step Time Asymmetry (%)**	F(1,13) = 2.040 p = 0.177		F(1,13) = 0.743 p = 0.404		F(1,13) = 0.753 p = 0.401	
